# The complete chloroplast genome sequence of precious tree, *Magnolia baillonii*

**DOI:** 10.1080/23802359.2019.1660248

**Published:** 2019-09-02

**Authors:** Jinfeng Zhang, Dejun Yang, Yi Wang

**Affiliations:** Laboratory of Forest Plant Cultivation and Utilization, Yunnan Academy of Forestry, Kunming, People's Republic of China

**Keywords:** *Magnolia baillonii*, chloroplast genome, precious wood

## Abstract

*Magnolia baillonii* is an important tropical and sub-tropical precious wood tree species in China. In this study, the complete chloroplast genome (cpDNA) sequence of *M. baillonii* was determined from Illumina HiSeq pair-end sequencing data. The cpDNA is 160,107 bp in length, contains a large single copy region (LSC) of 88,141 bp and a small single copy region (SSC) of 18,905 bp, which were separated by a pair of inverted repeat (IR) regions of 26,574 bp. The genome contains 132 genes, including 87 protein-coding genes, eight ribosomal RNA genes, and 37 transfer RNA genes. The overall GC content of the whole genome is 39.3%. Further phylogenomic analysis showed that *M. baillonii* clustered together with *Magnolia insignis*.

*Magnolia baillonii* Pierre [synonymous with *Paramichelia baillonii* (Pierre) Hu, *Micbelia baillonii* (Pierre) Fin. and Gagnep., *Aromadendron spongocarpum* (King) Craib., and *A. baillonia* (Pierre) Craib.] is fast growing and adaptable to a wide range of environmental and soil conditions (Figlar and Nooteboom 2004; Su et al. 2012). *Magnolia baillonii* is mainly distributed in China, India, Myanmar, Thailand, and Vietnam (Li et al. 2008). *Magnolia baillonii* has straight trunk and beautiful wood pattern. It is an important tropical and sub-tropical precious wood tree species in China. It is used in plywood, wood floor, sliced veneer, fine-grained high-quality furniture industries (Chen et al. 2011). It also produced anti-tumor constituents in its bark and has potential medicinal values (Ruangrungsi et al. 1987). However, there have been no reports on chloroplast genome information of *M. baillonii* until now. In this study, the complete chloroplast genome of was reconstructed based on the whole-genome Illumina sequencing to provide the underlying information for genetic and conservation studies and explore possible phylogenetic relationship within Magnolia genus.

The fresh leaf from a single individual of *M. baillonii* growing in Jinghong of China was collected (22°25′19″ N, 101°6′23″ E), and total genomic DNA was extracted using DNA Plantzol Reagent (Invitrogen, Carlsbad, CA, USA). The specimen of *M. baillonii* was deposited at Yunnan Academy of Forestry, Kunming, China and the accession number is YAFH0012653. High-throughput sequencing was carried out on the Illumina HiSeq system following the manufacturer’s protocol (Illumina, CA, USA; Hahn et al. 2013). Aligning, assembly, and annotation were conducted by CLC de novo assembler (CLC Bio, Aarhus, Denmark), BLAST, GeSeq (Tillich et al. 2017), and GENEIOUS v 11.0.5 (Biomatters Ltd, Auckland, New Zealand) by comparing with the genomes of *Magnolia insignis* (KY921716). To confirm the phylogenetic position of *M. baillonii*, other eighteen species of genus Magnolia from NCBI were aligned using MAFFT v.7 (Katoh and Standley 2013) and maximum likelihood (ML) bootstrap analysis was conducted using RAxML (Stamatakis 2006); bootstrap probability values were calculated from 1000 replicates. *Liriodendron chinense* (KU170538) was served as the out-group. The complete and annotated chloroplast genome sequence of *M. baillonii* has been submitted to GenBank with the accession number MK782763.

Paired-end reads were sequenced by using Illumina HiSeq system. In total, about 19.8 million high-quality clean reads were generated with adaptors trimmed. The complete *M. baillonii* plastid genome is a circular DNA molecule with the length of 160,107 bp, contains a large single copy region (LSC) of 88,141 bp and a small single copy region (SSC) of 18,905 bp, which were separated by a pair of inverted repeat (IR) regions of 26,574 bp. The overall GC content of the whole genome is 39.3%, and the corresponding values of the LSC, SSC, and IR regions are 37.9%, 34.3%, and 43.2%, respectively. The plastid genome contained 132 genes, including 87 protein-coding genes, eight ribosomal RNA genes, and 37 transfer RNA genes. The genome contained 92 unique genes; 20 genes duplicated in the IRs. Among annotated genes, seven (rps16, rpoC1, rpl2, petB, ndhB, ndhA, and atpF) contained a single intron. One copy of the rps12 gene harbored two introns and the second, incomplete and probably pseudogene copy had only one intron.

Phylogenetic analysis showed that *M. baillonii* clustered together with *Magnolia insignis* (Figure 1) which indicated the phylogenesis classification of *M. baillonii*. The determination of the complete plastid genome sequences provided new molecular data to illuminate the Magnolia evolution.

**Figure 1.**
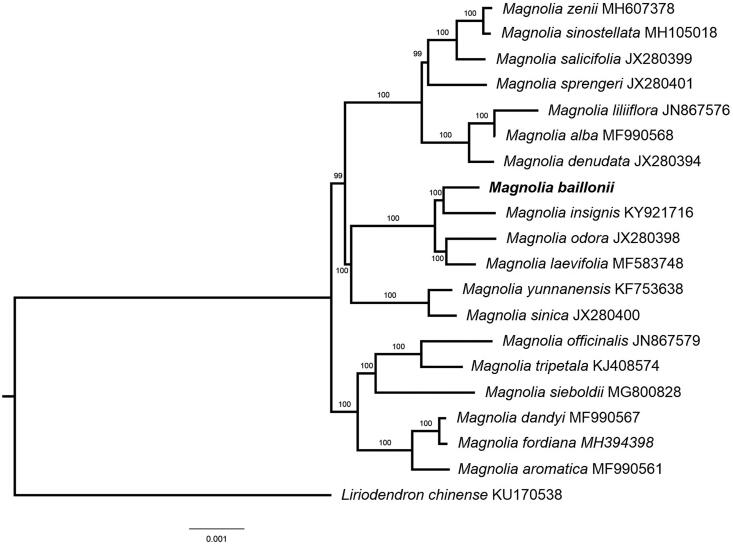
The maximum-likelihood tree based on the 19 chloroplast genomes of genus Magnolia. *Magnolia baillonii* is shown in bold and the bootstrap value based on 1000 replicates is shown on each node.
